# Nurse educators’ experiences of case-based education in a South African nursing programme

**DOI:** 10.4102/curationis.v38i2.1523

**Published:** 2015-12-09

**Authors:** Felicity M. Daniels, Lorraine P. Fakude, Ntombizodwa S. Linda, Regis R. Marie Modeste

**Affiliations:** 1 School of Nursing, University of the Western Cape, South Africa

## Abstract

**Background:**

A school of nursing at a university in the Western Cape experienced an increase in student enrolments from an intake of 150 students to 300 students in the space of one year. This required a review of the teaching and learning approach to ensure that it was appropriate for effective facilitation of large classes. The case-based education (CBE) approach was adopted for the delivery of the Bachelor of Nursing programme in 2005.

**Aim:**

The aim of the study was to explore nurse educators’ experiences, current practices and possible improvements to inform best practice of CBE at the nursing school in the Western Cape.

**Methods:**

A participatory action research method was applied in a two day workshop conducted with nurse educators in the undergraduate nursing programme. The nominal group technique was used to collect the data.

**Results:**

Three themes emerged from the final synthesis of the findings, namely: teaching and learning related issues, student issues and teacher issues. Amongst other aspects, theory and practice integration, as well as the need for peer support in facilitation of CBE, were identified as requiring strengthening.

**Conclusion:**

It was concluded that case-based education should continue to be used in the school, however, more workshops should be arranged to keep educators updated and new staff orientated in respect of this teaching and learning approach.

## Introduction and background

Case-based education (CBE) is an instructional design that involves interactive, student-centred exploration of realistic and specific situations (Uys & Gwele 2005). CBE refers not only to real-life patient cases but can include fictional, virtual or simulated cases (Thistlethwaite *et al*. 2012). According to Williams (2005), CBE includes the use of problems or cases to stimulate learning and for the acquisition of knowledge, skills and attitudes. For nursing, this means that constructed cases link learning with the reality in clinical practice.

A case may be classified according to the level of complexity depending on the level of the students’ education; or according to its duration as a single educational intervention, covering a specific learning issue; or a longitudinal approach that spans over time, with the possibility of developing the case into steps that are appropriate according to the year level in a learning programme (Bowe, Voss & Thomas Aretz 2009). Specific cases are carefully designed by educators, based on the specific outcomes of the module or course. The purpose is to allow students to actively engage with ‘real world’ issues. This encourages students to solve problems in class, which are similar or mimic those that occur in the real world and could subsequently advance active learning, critical thinking, communication skills, and other professional competencies (Bowe *et al*. 2009). Problem solving in a classroom rather than in a real life setting also provides a safe environment in which students can learn.

The national imperative to improve the status of higher education in South Africa is embedded in the Department of Education’s (DoE) National Plan for Higher Education (DoE 2001) which gives effect to the vision for the transformation of the higher education system as outlined in the Education White Paper 3 (DoE 1997). One of the goals of the National Plan for Higher Education is:

to provide increased access to higher education to all irrespective of race, gender, age, creed, class or disability and to produce graduates with the skills and competencies necessary to meet the human resource needs of the country. (DoE 2001:17)

The National Plan also proposes computer literacy, information management, communication and analytical skills as important skills and competencies with which all graduates should be equipped, in order to function in a modern society.

Another goal of the National Plan for Higher Education is:

to promote equity of access and to redress past inequalities through ensuring that the staff and student profiles in higher education progressively reflect the demographic realities of South African society. (DoE 2001:17)

In order to meet these priorities, schools and departments at higher education institutions had to restructure their educational programmes offerings on a number of levels.

At the school of nursing, restructuring included increasing the intake of nursing students, and increasing the number of lecturers and clinical supervisors accordingly. Also in line with the increased intake of students, a review of various applicable teaching strategies was undertaken and, finally, a decision was taken to adopt CBE as the teaching and learning strategy to drive the Bachelor of Nursing programme. CBE was selected based on its potential to promote active participation and the development of critical, analytical and problem solving skills of nursing students. Nurse educators should be able to implement innovative teaching and learning strategies such as CBE in classes with a large number of students. However, their experience of facilitating CBE should be explored in order to identify strengths that could be enhanced and weaknesses that should be addressed.

## Problem statement

National transformation of higher education in South Africa occurred not only in terms of ensuring programme enhancements, but also in terms of institutional mergers and programme collaborations across institutions during the early 2000s. Decisions regarding nursing education in the Western Cape ultimately led to only two universities being identified as the enrolling institutions for the undergraduate nursing programme, which would be offered in collaboration with the other two universities in the province. As a result of the nurse shortage in the Western Cape Province, a substantial increase in the student nurse enrolment targets was planned for the two enrolling institutions (DoE 1997).

The two nursing departments were tasked to review and strengthen their undergraduate programmes, develop and foster collaboration between higher education institutions in the region and ensure that the students would have a positive learning experience. This resulted in the submission of revised programmes to the South African Nursing Council. CBE was adopted as the teaching and learning approach for the revised undergraduate nursing programme offered at one of the universities since 2005. The programme was phased in gradually over a period of four years whilst the existing programme at the time was phased out.

A study conducted by Daniels and Khanyile in the Western Cape in 2010, found that nurse educators were dissatisfied with the use of CBE; and that challenges in respect of substantial staff turnover were experienced, as many members of the faculty were employed on a contract basis rather than in permanent positions. The high staff turnover necessitated continual updating of existing staff and orientation of new staff to CBE. In addition to the study by Daniels and Khanyile (2010), students’ evaluations revealed inconsistencies in the facilitation of CBE in the various year levels of the programme, as well as in the subject disciplines. Nurse educators who facilitate CBE in the school of nursing reflected on students’ feedback and confirmed that the abovementioned was congruent with their own experiences. After six years of implementation, it became necessary to explore experiences and the current practices related to the implementation of CBE and to establish possible ways of improving on its implementation, if necessary.

### Literature review

Educators who are involved in nursing education strive to enhance the learning process of the students, to ensure the preparation of nurses who are ready to practice as required in their place of work. CBE has been documented as one of the approaches that facilitates learning (Sangestani & Khatiban 2013).

Effective application of a case-based teaching and learning process requires high quality cases that facilitate higher-order learning and competent teachers who support students during the teaching and learning process (Ramaekers *et al*. 2011). CBE enhances the use of critical thinking, as suggested by Bowe *et al*. (2009), by providing opportunities to students for examining the cases under discussion and identifying emerging problems or issues presented in the case studies.

DeSanto-Madeya (2007:324) states that developing teaching strategies that can improve skills such as decision making, critical thinking and problem solving is a continuous challenge for nurse educators. In order to attain these skills, changes need to take place in the teaching and learning environment. This would be more feasible with student-centred rather than teacher-centred teaching and learning strategies. Another advantage of CBE is the potential for health professionals to develop clinical reasoning skills as it links theory to practice (Thistlethwaite *et al*. 2012).

CBE is not a new teaching and learning approach, and has been implemented in different fields of learning. As noted by Uys and Gwele (2005), CBE has many benefits, such as facilitating the students’ active participation whilst providing real life situations to which theoretical knowledge is applied. Additionally, by using group work, CBE provides many opportunities for creativity, collaborative work, and integration of socio-cultural aspects of health care to be included in teaching and learning interventions. The students’ challenges are addressed by the educators and debriefing is undertaken when students talk about daily life issues. At the same time reflection, communication and decision making skills are being enhanced (Uys & Gwele 2005).

A case, as applied in CBE, is either a real, semi-fictional or a fictional depiction of a situation that requires some type of decision making, and may be descriptive or narrative in nature. When using case studies in teaching and learning, the desired results include critical and creative thinking, active participation of learners, and the application of theory to practice, as referred to earlier. Effective cases encourage and facilitate integrative learning where a number of modules from different disciplines could be combined. In professions such as nursing, cases ensure the correlation of theory and practice, as the management of the case requires evidence of both theoretical and clinical enquiry (DeSanto-Madeya 2007; Sangestani & Khatiban 2013).

From the students’ perspective, a case-based approach has been noted to provide more opportunity in the preparation of students for deep learning compared to the lecture-based approach (Harman *et al*. 2015; Ilgüy *et al*. 2014). Studies have found that the use of CBE benefited students, as they scored higher on assessment tasks that required analysis and evaluation and on patient assessment, diagnosis and treatment plans (Ilgüy *et al*. 2014; Raurell-Torredà *et al*. 2015). Furthermore, it was found that students were better at integration of theory and practice (Hansen *et al*. 2005; Harman *et al*. 2015); and developed independent learning, problem solving and critical thinking skills, with the opportunity to think more broadly (Harman *et al*. 2015).

The application of CBE has been noted to improve the learning process, as group participation and collaboration are enhanced (Harman *et al*. 2015). Achieving group participation and collaboration, as an outcome of the learning process, has been found to be difficult in traditional teaching and learning approaches. CBE has also been reported by students as being enjoyable and interesting (Hansen *et al*. 2005; Williams 2005).

The application of the case-based teaching and learning approach at the school of nursing in the Western Cape follows four steps, namely:

1. reviewing the case
2. data gathering
3. analysis of the solution
4. presentation of findings.

As co-operative learning is applied during the teaching and learning process, students are encouraged to meet outside class sessions for preparation before case presentations.

## Aim of the study

The aim of the study was to explore nurse educators’ experiences, current practices and possible improvements to inform the best practice of CBE at the nursing school in the Western Cape.

### Significance of the study

The results of this study have the potential to assist with developing strategies aimed at enhancing the teaching and learning of student nurses, and more specifically with the implementation of CBE in the circumstance of a large number of students. An improved approach to implementing CBE, based on the results of this study, may improve the alignment of theory to practice. It is anticipated that by improving student learning, patient outcomes will improve, as students would have developed the skills to apply critical judgement in patient care.

## Research methods and design

**FIGURE 1:**
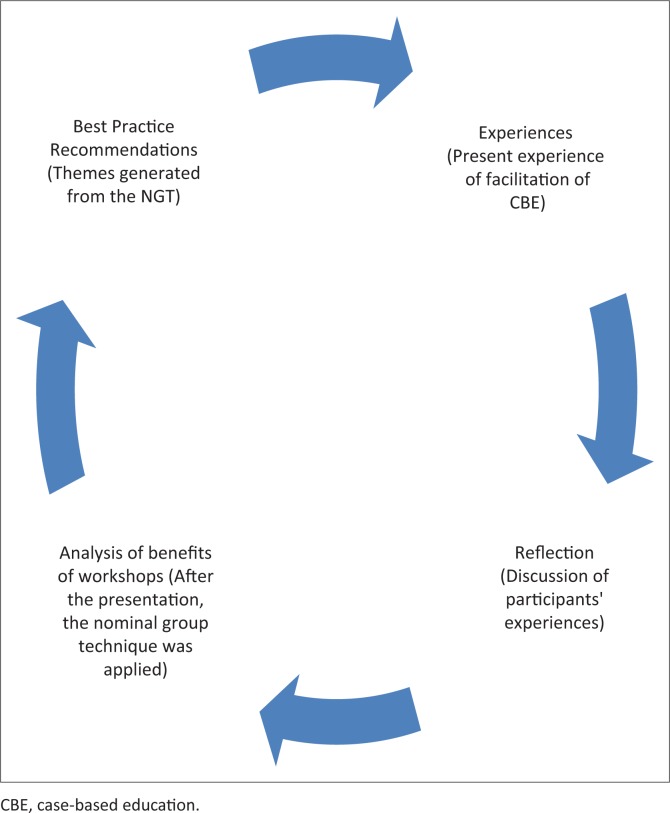
Application of the action cycle.

A two day workshop was held in 2012 and a participatory action research design was applied, as it provided an opportunity for participant collaboration and reflection (Baum, MacDougall & Smith 2006). A participatory action research cycle was followed consisting of the steps of reflecting on present experiences, a reflective discussion, an analysis of the benefits of the workshops and best practice recommendations. As reflected in Figure 1, the selection of a participatory action research methodology was most appropriate, resulting from its ability to amalgamate action and reflection, as well as theory and practice (Baum *et al*. 2006).

### Population and sampling

The population and sample comprised all nurse educators (nursing lecturers and clinical supervisors) who facilitated theoretical and clinical teaching and learning in the Bachelor of Nursing programme at the school of nursing at a university in the Western Cape. All nurse educators (nursing lecturers and clinical supervisors) were invited to participate. A total of 40 nurse educators voluntarily agreed to participate and attended the workshop.

### Data collection

On day one of the workshop, participants were asked to capture in writing their current *experience* and practice of facilitating learning using CBE. The question was posed, ‘What are the current experience and practice in CBE at the school of nursing?’ This was the first step in the action research cycle. The participants’ personal exploration of their experiences highlighted their strengths and challenges in terms of facilitation of CBE.

The second step of the action research cycle required participants to *reflect* on and share their experiences in a group *discussion*.

A presentation on case-based education followed the above two steps. Thereafter, participants were divided into Group A and Group B for the remainder of the two days of the workshop. One group was formed by nurse educators from year one and two of the Bachelor of Nursing programme and the second group comprised nurse educators from the third and fourth year.

Based on the participants new understanding of CBE, a second question was posed, ‘What do you view as the most important practices that we, as nurse educators should adopt as best practice in CBE at the school of nursing?’ This required participants to reflect on practices of CBE in the school of nursing. The nominal group technique (NGT) process was used in the discussion and analysis of the second question and included the following steps, documented by Potter, Gordon and Hamer (2004):

- introduction and explanation
- silent generation of ideas
- sharing ideas
- group discussion
- voting
- ranking.

This was the third step of the action research cycle.

The last step of the action research cycle involved the identification of *best practice* for the facilitation of CBE. These emerged as themes from the NGT process.

### Data analysis

The data gathered from the discussion of the first question were analysed qualitatively using thematic content analysis and were presented with the aim of emphasising the participants’ experiences of the use of CBE. During the thematic content analysis, themes and subthemes were extracted from the documented experiences of the participants, providing a clearer understanding of the experiences. The data from a NGT were collated and ranking for each group was presented.

## Results

There were different levels of understanding and practice amongst nurse educators with regard to the case-based method of teaching. Expressed learning needs or expectations of the workshop varied according to the year level of teaching, as well as to the specific category of lecturers and clinical supervisors. This section presents, firstly, the results from the nurse educators’ discussions about their current practices and challenges in the implementation of CBE and, secondly, the developed themes from the NGT discussions which are the improvement plans and best practices.

### Current practices of case-based education

The participants reported using a number of strategies in the implementation of CBE, which maximised the benefits of CBE – such as group work, self-study and reflection. They reported that CBE extended beyond theoretical learning in the classroom to include the learning of clinical skills in the clinical settings outside the classroom. One of the participants affirmed that:

‘the use of different real cases facilitate varied learning opportunity, as this is viewed as good in assisting the students in comprehension of the realities of life out there. (Group B)

Both lecturers and clinical supervisors are involved in the accompaniment and clinical supervision of students. However, although the lecturers conduct clinical assessments, the bulk of clinical assessments are undertaken by the clinical supervisors. Clinical teaching and learning for Bachelor of Nursing students occurs in simulation laboratories and during placement for clinical practice. Most responses from the participants related to the clinical placement environment where active, meaningful learning took place, and which supplemented the learning that had taken place in class. In the clinical settings students, as adult learners, learn most of their skills independently. In the skills laboratory, a very structured method called the skills lab method (SLM) is followed. As noted by Jeggels, Traut and Kwast (2010), the SLM is an innovative strategy used by the school of nursing in the Western Cape for clinical skills training, in well-equipped clinical skills laboratories, using simulated patients.

The SLM is complemented by real life experience in clinical practice, where the educators assume responsibility to accompany and work side-by-side with the student (Group B). They encouraged the students to participate in the doctor`s ward rounds by presenting patient cases, as noted by one participant:

‘By encouraging the students to read available material [*documents*] at the clinical site, it promotes problem-solving skills allowing the students to provide patient-centred care, skills that enhance the application of CBE as applied in the programme.’ (Group B)

In health professions education, the integration and application of theory and practice is fundamental to the development of clinical competence. Participants reported implementing strategies to enhance the integration of theory and practice as indicated by one participant:

‘In the skills lab, the facilitators [*lecturers and clinical supervisors*] focus on determining students’ pre-knowledge about particular skills that will be demonstrated and integration with theory to simulate real life scenarios.’ (Group B)

Group work is one of the major teaching techniques used in theoretical as well as clinical teaching and learning situations. It allows students to support one another in developing the necessary skills for future application with a variety of health care professionals in the clinical setting. Additionally, simulated patient encounters (SPEs) are used for the development of clinical and communication skills and for enhancing integration of theory and practice. Participants also reported using several other techniques, including but not limited to sharing their own experiences with each other, by referring to their own real life experiences.

During the tutorial groups, or clinical placement, students’ challenges were addressed either by reflection and self-discovery, or by a restorative function, during which the educators played a supportive role. Reflection is in line with the foundational philosophy of CBE (Uys & Gwele 2005). In addition to class discussions, the educators provide lecture input to assist the students to apply their knowledge and improve integration of the content. This occurred at opportune times during the CBE process, but most often it is undertaken at the end of the class contact session.

### Implementation challenges

One of the challenges reported by the participants was related to time constraints for the educators and students themselves. Participants acknowledged that despite group facilitation being the main technique and a core responsibility of the educator, when facilitating student learning, they reported that they often did not have enough time to facilitate this process as well as they would have wanted to, resulting from a lack of time and the ‘packed’ learning programme:

‘The students are thus encouraged to meet in their groups outside of the classroom [*session*].’ (Group B)

This is undertaken after or before class sessions as is characteristic of CBE (Uys & Gwele 2005). Educators encourage students to take responsibility for their learning as self-directed learners and to strengthen the collaboration with group members. It is, however, often challenging for students to meet as a group, resulting from their workload.

Another challenge identified by the participants was the students’ limited pre-knowledge which inhibited effective learning; in addition to limited effort which some students demonstrated when they read word for word from the textbook when presenting a case (Group A). Furthermore, it was reported that the educator’s skills in facilitating learning within CBE was not always adequate; one of the clinical supervisors reported that at times one did not know whether or not educators and students ‘were on the same page’ (Group B). In this regard, it was reported that some educators were not skilled to guide the students in the process of discovery. Assessment strategies of this approach were also reported as a challenge by the majority of the nurse educators.

### Further plans for improvement and best practice

The second part of the workshop used the NGT to collect data related to the best practices that can be adopted in CBE at the school of nursing. Seven items emerged from the data analysis of the nominal group technique process as reflected in Table 1. Both groups identified integration of theory and practice as the most important component that should be adopted in the implementation of CBE. They also identified the importance of the facilitation process as being crucial to successful CBE. The participants’ feedback indicated that different participants had a different understanding of their teaching practice of CBE. Although the most important item was the same for both groups, it was noted that only two of the seven items were similar for both groups. It was also noted that both groups identified themes related to the educator whilst only group two identified aspects related to student (Table 1).

**TABLE 1:** Ranked themes per group, horizontal and core themes.

Group 1 vertical themes	Group 2 vertical themes	Horizontal themes	Core themes
Integration of theory and practice	Integration of theory and practice	Integration of theory and practice	Teaching and learning issues
Personal and professional development	-	Facilitator issues	Facilitator issues
Facilitation of learning (process of learning)	Facilitation process	Facilitation	Teaching and learning issues
Practice issues (setting boundaries)	Facilitator’s working environment	Facilitator issues	Facilitator issues
-	Selection of students	Student issues	Student issues
-	Assessment	Assessment	Teaching and learning issues

**FIGURE 2:**
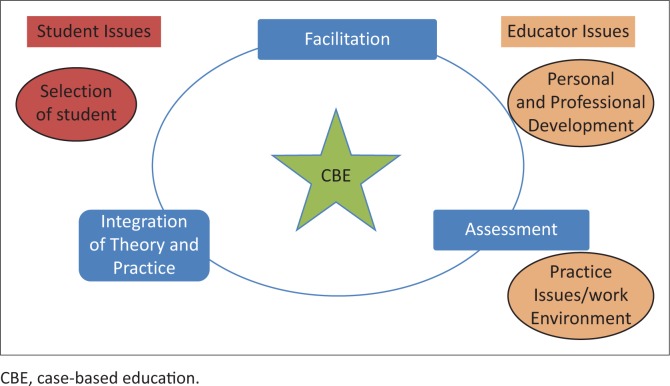
Synthesis of findings.

The vertical themes of Groups 1 and 2 are ranked based on the NGT priority listing. The horizontal themes represent the similarity of themes across the two groups whilst maintaining the ranking. The core themes are the outcome of further synthesis of the horizontal themes, as reflected in Figure 2. The synthesised core themes include teaching and learning related issues, student issues and educator issues.

### Theme 1: Teaching and learning issues

The responses of the participants pertained to activities and tasks that participants regarded as their responsibilities as educators. Those responsibilities included the role of a nurse educator (lecturers and clinical supervisors) in managing the process of learning in the CBE approach. The participants’ experiences related to both theory and clinical teaching. The most important aspects that were identified by both groups in terms of the teaching and learning session in CBE were:

- facilitation
- integration of theory and practice
- assessment.

#### Facilitation activities

Participants reported that activities such as role play, facilitation of small groups, interactive group discussions and presentations should be used to assist students with integrating a relevant case to particular subject matter (Group B). Most participants regarded activities such as dividing the students into small groups, explaining the pre-requisite knowledge, identifying appropriate resources, and assisting the students to gather required resources for their presentations as important.

#### Integration of theory and practice

Participants highlighted that it is important to guide students to understand the specific outcomes of every contact session (Group B) and provide triggers or questions about the case. During students’ presentations, links to real life experiences would be pointed out for students to note, which enhances integration of theory and practice as noted by one participant:

‘I encourage active learning of student[*s*] in analysing the situation [*case*] by seeking [*the*] student to cooperate and provide feedback; I evaluate the student to establish if the student comprehends the case. I believe this actually stimulates critical thinking in the student.’ (Group B)

#### Assessment

Having identified assessment as a challenge in the implementation of CBE at the school of nursing, it was noted that to further improve the implementation of CBE, additional workshops were needed with a specific focus on assessment strategies.

### Theme 2: Educator issues

In the teaching and learning process, the role of the educator is crucial, and in this study, the issues related to the educator, which potentially affected effective implementation of CBE, were identified as *personal and professional development, and practice issues and work environment*.

#### Personal and professional development

During discussions, the participants emphasised the importance of being knowledgeable about the subject or content that was taught, as well as the CBE facilitation process, as it influenced the educator’s ability to competently manage the learning process. It is, therefore, important to strengthen these educator related aspects in order to enhance the teaching and learning process. Ramaekers *et al*. (2011) noted that one of the important conditions for CBE is having competent teachers who support students throughout the teaching and learning process.

#### Practice issues and work environment

The educators agreed that the work environment where they functioned should be supportive and that there should be frequent in-service sessions or workshops on CBE.

### Theme 3: Student issues

The third aspect to be considered for effective implementation of CBE was the *selection of students* into the programme. It was mentioned by participants that the capabilities of students accepted into the programme should be carefully considered as they would need to cope in a CBE environment. The participants proposed that the students who were selected for the programme should be motivated and able to engage in self-dircted learning, as these are criteria for effective CBE.

## Discussion

Participants in the study highlighted various strategies that are employed in the implementation of CBE at the specific nursing school. These included the use of group work, reflection and student presentation. This is consistent with what has been reported in literature on the use of CBE as a teaching and learning approach (Harman *et al*. 2015; Raurell-Torredà *et al*. 2015; Williams 2005). As noted by Harman *et al*. (2015) and Ramaekers *et al*. (2011), the educator is responsible for facilitating learning and for providing opportunities for students to develop critical skills. In this study, the participants reported being challenged by inadequate skills to facilitate learning in CBE. This was highlighted as important for best practice. The participants indicated the need to strengthen their facilitation skills through further in-service training sessions. Improving the competence of the educators to facilitate CBE will, thus, improve student learning (Williams 2005) as students are better supported by competent teachers (Ramaekers *et al*. 2011).

Similar to previous studies such as Harman *et al*. (2015), time constraints were noted by participants as being a challenge. Furthermore, the pre-knowledge of students was reported to be limited, hence the emphasis on the caliber of the students to be recruited and selected in the programme. However, this limitation could easily be linked to a lack of organisational skills and accountability on the part of the student, as was noted by Harman *et al*. (2015). Students in higher education are expected to use higher-order thinking and take responsibility for their own learning. It is important, therefore, that these skills are developed by students from an early stage in the programme. The educators should strive to develop students who are independent and responsible for their own learning (Harman *et al*. 2015).

## Recommendations

As part of the last step of participatory action research applied in the study, participants determined the follow up actions required. It was agreed that the educators would continue to learn more about CBE and strive towards improving their facilitation of CBE. Future planned activities were recommended to strengthen the implementation of CBE at the school in question, and included:

- follow-up workshops to ensure that what has been learnt would be retained
- specific workshops to be conducted which focus on facilitation and assessment relevant to CBE.

It is anticipated that information and skills gained in these workshops will enhance the implementation of CBE in the school.

It was agreed that the cases presented in the student’s module guides required revision. Furthermore, peer support in relation to CBE facilitation was proposed, which could be undertaken by visiting each other’s classroom sessions and providing feedback. It was also suggested that starting with a simple and well-structured case study, and gradually adding complexity by introducing more contextual features and structure, as seen with problem-based learning (PBL), seems to be a logical way to slowly transition students from a predominantly dependent student mode to more autonomous learners who are ready for practice. Educators should challenge students to develop clinical judgment and problem-solving skills, by asking students the ‘why’ questions, where appropriate.

## Ethics statement

The workshop presented in this paper was conducted as part of an ethically approved broader study which evaluated the Common Teaching Platform for undergraduate nursing in the Western Cape, and aimed at improving the offering of the Bachelor of Nursing programme (Registration No. 10/4/24). The participants were informed about the purpose of the workshop and the intent to publish its outcomes; they also consented voluntarily to participate after the process was explained to them. Anonymity and confidentiality were maintained, by ensuring that the participants’ names were not revealed. Data collected in the study were only available to the researchers, and kept safely throughout. Participants’ comfort was ensured by providing an appropriate venue.

### Academic rigor

In order to increase the rigor of the study and the qualitative data collected, trustworthiness was established by ensuring confirmability, dependability, transferability, and credibility of the data and findings (Creswell 2014). Some of the strategies employed to bolster the trustworthiness of the study were: member checking, debriefing, source triangulation, and auditing of the analysis by cross checking from the authors.

### Limitations of the study

This study was conducted at one institution that offers an undergraduate nursing programme in South Africa. The findings of the study cannot be generalised as this was conducted as a qualitative study and was contextual in nature. However, a detailed description of the study procedure provides an opportunity for the study to be replicated in other settings.

## Conclusion

The workshop proceedings served three purposes. Firstly, it allowed the collection of data from the participants with regard to their views of current practices in CBE and, secondly, it allowed the participants to identify the best practices to be adopted by the school of nursing. Thirdly, presentation and active discussion were part of staff capacity development, with regard to each individual’s report of his or her experience of his or her own practice in relation to colleagues in the same discipline, year level, or immediate context. This is part of the development as it identified ways of improving one’s practice as an educator facilitating CBE. This aspect of staff development is essential, as the educator’s preparation for the new role is crucial for success (Uys & Gwele 2005).
